# A four-decade analysis of the incidence trends, sociodemographic and clinical characteristics of inflammatory bowel disease patients at single tertiary centre, Kuala Lumpur, Malaysia

**DOI:** 10.1186/s12889-019-6858-2

**Published:** 2019-06-13

**Authors:** Norfilza Mohd Mokhtar, Khairul Najmi Muhammad Nawawi, Jaarvis Verasingam, Wong Zhiqin, Ismail Sagap, Zairul Azwan Mohd Azman, Luqman Mazlan, Hamzaini Abdul Hamid, Nur Yazmin Yaacob, Isa Mohamed Rose, Eden Low Ngah Den, Mah Suit Wan, Raja Affendi Raja Ali

**Affiliations:** 1 Department of Physiology, Faculty of Medicine, UKM Medical Centre (UKMMC), Kuala Lumpur, Malaysia; 2 Gastroenterology Unit, Department of Medicine, UKM Medical Centre (UKMMC), Kuala Lumpur, Malaysia; 3 Colorectal Unit, Department of Surgery, UKM Medical Centre (UKMMC), Kuala Lumpur, Malaysia; 4 Department of Radiology, Faculty of Medicine, UKM Medical Centre (UKMMC), Kuala Lumpur, Malaysia; 5 Department of Pathology, Faculty of Medicine, UKM Medical Centre (UKMMC), Kuala Lumpur, Malaysia; 6 Department of Pharmacy, Faculty of Medicine, UKM Medical Centre (UKMMC), Kuala Lumpur, Malaysia

**Keywords:** Inflammatory bowel disease, Crohn’s disease, Ulcerative colitis, Incidence, Prevalence

## Abstract

**Background:**

Inflammatory bowel disease (IBD) was once considered as a Western disease. However, recent epidemiological data showed an emerging trend of IBD cases in the Eastern Asia countries. Clinico-epidemiological data of IBD in Malaysia is scarce. This study aimed to address this issue.

**Methods:**

Retrospective analysis of ulcerative colitis (UC) and Crohn’s disease (CD), diagnosed from January 1980 till June 2018 was conducted at our centre.

**Results:**

A total of 413 IBD patients (281 UC, 132 CD) were identified. Mean crude incidence of IBD has increased steadily over the first three decades: 0.36 (1980–1989), 0.48 (1990–1999) and 0.63 per 100,000 person-years (2000–2009). In the 2010 to 2018 period, the mean crude incidence has doubled to 1.46 per 100,000 person-years. There was a significant rise in the incidence of CD, as depicted by reducing UC:CD ratio: 5:1 (1980–1989), 5:1 (1990–1999), 1.9:1 (2000–2009) and 1.7:1 (2010–2018). The prevalence rate of IBD, UC and CD, respectively were 23.0, 15.67 and 7.36 per 100,000 persons. Of all IBD patients, 61.5% (*n* = 254) were males. When stratified according to ethnic group, the highest prevalence of IBD was among the Indians: 73.4 per 100,000 persons, followed by Malays: 24.8 per 100,000 persons and Chinese: 14.6 per 100,000 persons. The mean age of diagnosis was 41.2 years for UC and 27.4 years for CD. Majority were non-smokers (UC: 76.9%, CD: 70.5%). The diseases were classified as follows: UC; proctitis (9.2%), left-sided colitis (50.2%) and extensive colitis (40.6%), CD; isolated ileal (22.7%), colonic (28.8%), ileocolonic (47.7%) and upper gastrointestinal (0.8%). 12.9% of CD patients had concurrent perianal disease. Extra intestinal manifestations were observed more in CD (53.8%) as compared to UC (12%). Dysplasia and malignancy, on the other hand, occurred more in UC (4.3%, *n* = 12) than in CD (0.8%, *n* = 1). Over one quarter (27.3%) of CD patients and 3.6% of UC patients received biologic therapy.

**Conclusion:**

The incidence of IBD is rising in Malaysia, especially in the last one decade. This might be associated with the urbanization and changing diets. Public and clinicians’ awareness of this emerging disease in Malaysia is important for the timely detection and management.

## Background

Inflammatory bowel disease is a chronic condition, characterized by relapsing and remitting inflammation of gastrointestinal (GI) tract. It encompasses Crohn’s disease [1] (CD), which can affect any segment of GI tract, and ulcerative colitis (UC), that involves exclusively the rectum and colon. Although UC and CD share a number of similar clinical features, each does have distinct intestinal manifestation [2]. Patients with UC and colonic CD most commonly present with chronic diarrhoea, per rectal bleeding and accompanied by abdominal pain. On the other hand, ileocolonic CD mainly manifests as abdominal pain localized at periumbilical or right lower quadrant, with or without watery diarrhoea. Vague abdominal pain might be the only symptom for small bowel CD, though more extensive small bowel involvement will cause postprandial abdominal pain, nausea, vomiting and watery diarrhoea. In contrast to UC, perianal disease such as perianal abscess, fistula and fissure can occur in CD [3]. Extra intestinal features for IBD include fever, weight loss, arthralgia, mucocutaneous lesions such as oral ulcers, erythema nodosum, pyoderma gangrenosum and ophthalmologic complications like episcleritis, iritis and uveitis [3].

At present, there is no cure for IBD and therefore the management is aimed at induction and maintenance of the disease remission. Due to its chronicity, IBD can results in significant long-term morbidity, impairment of patient’s health-related quality of life and excess health care resource use. Study by Graff LA et al. revealed IBD patients with active disease had higher levels of distress, health anxiety, perceived stress, lower social support and poorer disease-specific quality of life as compared to those with inactive disease [4]. Longobardi T el al. examined the health care resource utilization by patients captured in the University of Manitoba IBD database. They reported that IBD patients compared with healthy controls were more likely to have an outpatient visit (RR, 1.18; CI, 1.17–1.19) and an overnight hospital stay (RR, 2.32; CI, 2.16–2.49) [5]. When examining the financial burden of the disease in Canada, a country with the highest prevalence and incidence rates of IBD in the world, Rocchi A et al. documented an estimated total cost of $2.8 billion in 2012 ($12,000 per IBD patient); with the direct medical costs exceed $1.2 billion and the indirect costs were dominated by long-term work losses of $979 million [6].

IBD was once considered a Western disease. Based on a systematic review in 2012, the highest annual incidence of IBD was recorded in Europe (UC: 24.3 per 100,000 person-years, CD:12.7 per 100,000 person-years), followed by North America (UC: 19.2 per 100,000 person-years, CD: 20.2 per 100,000 person-years) and Asia plus the Middle East (UC: 6.3 per 100,000 person-years, CD: 5.0 per 100,000 person-years) [7]. While the IBD incidence rates in Western countries has remained relatively stable or steadily increased over time, however it is a rapid rise among Asian countries. For instance, a population-based study from South Korea showed a 10-fold increase in the incidence of IBD over two decades (UC: 0.34 to 3.08 per 100,000 person-years, CD: 0.05 to 1.34 per 100,000 person-years) [7]. This epidemiology shift was likely to be caused by urbanization and changing dietary pattern towards Western diet, together with increased disease awareness and improved diagnostic tools [8]. Additionally, regular dining outside, high use of food flavouring and preservatives were among risk factors for developing colorectal cancer among Malaysian, which is one of the long term complication of IBD [9].

Locally, IBD is perceived as a rare disease and therefore its incidence, clinico-epidemiological and sociodemographic data in Malaysia are scarce. Malaysia’s annual incidence of IBD was reported as 0.94 per 100,000 person-years [10]. The first Malaysian study on incidence and prevalence of IBD by Hilmi et al. published in 2015, revealed the crude incidence of IBD was 0.68 per 100,000 person-years. In addition, the trend of IBD incidence was increasing over the past two decades (0.07 to 0.69 per 100,000 person-years) with it being the highest among the Indians (1.91 per 100,000 person-years) [11]. A more recent study from Southern Peninsular Malaysia (state of Johor) published in 2018 by Pang et al., showed a comparable result with the crude incidence of IBD was 0.68 per 100,000 person-years (UC: 0.27 per 100,000 person-years, CD: 0.36 per 100,000 person-years) [12].

The aim of this study was to determine the time trends of the incidence of IBD over the last four decades at a tertiary referral hospital, Universiti Kebangsaan Malaysia Medical Centre (UKMMC), Kuala Lumpur, Malaysia. This study was also observed at the sociodemographic and clinical characteristics of this IBD cohort. We hypothesized that there was an increasing trend of the IBD incidence at our centre for the last four decades, which reflected the overall incidence in Malaysia.

## Methods

### Study design and data collection

We performed a retrospective analysis on all IBD patients who was treated under gastroenterology and colorectal surgery units in UKMMC from January 1980 to July 2018. Data was collected from UKMMC IBD registry, patients’ medical records, hospital online information system and during follow up review. The UKMMC IBD registry is a prospectively maintained database that was initiated in 2013. All data prior to 2013 were collected retrospectively and added into the registry. It aimed to capture all the relevant information related to IBD patients who are treated in UKMMC which include sociodemographic details (age at diagnosis, gender, ethnicity, smoking status, education level and family history), disease characteristics (patients’ symptoms, Montreal’s classification, presence of extra-intestinal manifestation and disease complications), investigation results (blood tests, stool tests, radiology, endoscopy and histology) and treatment modalities (medical and surgical treatments). The data were collected from the patients directly, patients’ medical records, and hospital online information system. The collected data were stored in electronic spreadsheet and managed by the gastroenterology team members. This IBD registry is kept confidential, not accessible to the public and being updated regularly every 1–3 months. The quality control of the database was maintained by random checking handled by two independent medical staff and further validated by the Head of Gastroenterology unit. UKMMC is one of the four university teaching hospitals in Malaysia and located in Cheras, Kuala Lumpur. Kuala Lumpur is the capital of Malaysia and it covers an area of 243 km^2^. It has estimated population of 1.79 million in 2017 with population density of 7670 people per sq. km of land area. This tertiary hospital was founded in 1997, has 36,000 admissions per year and covers an urban multi-racial population in the Klang Valley. Malay and Chinese are the two major ethnic groups in Kuala Lumpur (47.2 and 41.4% respectively) followed by Indian, 10.2% and others, 1.2% [13]. It provides full gastroenterology service which include the inpatient, outpatient and endoscopy services.

### Diagnosis of inflammatory bowel disease

Diagnosis of IBD requires combined assessment of clinical signs and symptoms, blood tests (such as haemoglobin level, platelet level and inflammatory markers; erythrocyte sedimentation rate, and C-reactive protein, endoscopic findings, histopathological findings and relevant imaging such as computed tomography of abdomen/pelvis and magnetic resonance imaging (MRI) of small bowel and/or pelvis. All the IBD diagnosis was made by UKMMC gastroenterologists, after considering all the available diagnostic information. Any patients who did not meet the criteria of IBD were excluded from the analysis. By using Montreal’s classification, UC was classified according to disease location while CD according to disease location as well as disease behaviour.

### Incidence trend

The incidence trend of IBD, UC and CD was determined by comparing their mean crude incidence in each of the last four decades, i.e. 1980–1989, 1990–1999, 2000–2009 and 2010–2018. Population data (together with the average annual population growth rate) of Kuala Lumpur were obtained from the Department of Statistics, Malaysia and used as the denominator. The mean crude incidence was expressed as number of cases per 100,000 person-years.

### Prevalence

The prevalence of IBD, UC and CD was calculated based on the whole Kuala Lumpur population in 2018 and expressed as number of cases per 100,000 persons. Population data of Kuala Lumpur stratified by ethnicity (Malay, Chinese and Indian) in 2018 were also obtained from Department of Statistics, Malaysia. These data were used to calculate the prevalence of IBD stratified by ethnicity.

### Statistical analysis

The data were compiled and analysed using IBM SPSS Statistics version 24.0 (IBM Corporation, New York, USA). Continuous variables that were collected included age at diagnosis and duration of disease. Majority of data were summarized into categorical variables that included gender, ethnicity, smoking status, education level, family history positivity, major comorbidity, duration of disease, age group at diagnosis, disease location and behaviour, presence of extra-intestinal manifestation and disease complications, treatments received and type of surgery. Continuous variables were presented, according to a parametrical distribution, as mean and standard deviation. Categorical variables were presented as absolute value and percentage. Pearson’s chi square and one-way ANOVA test were used for the analysis of clinical characteristics. The duration of disease of IBD patients was stratified as follows: less than 5 years (< 5) was labelled as short disease duration; 5 to 10 years (5–10) was considered as long disease duration; and more than 10 years (> 10) was labelled as very long disease duration. The age of IBD diagnosis was classified as: adolescence if less than 19 years-old; young adults were between 19 to 35 years-old; middle-aged adults were between 36 to 55 years-old; and older adults were those above 56 years-old.

## Results

### Incidence trend of IBD

**Fig. 1:**
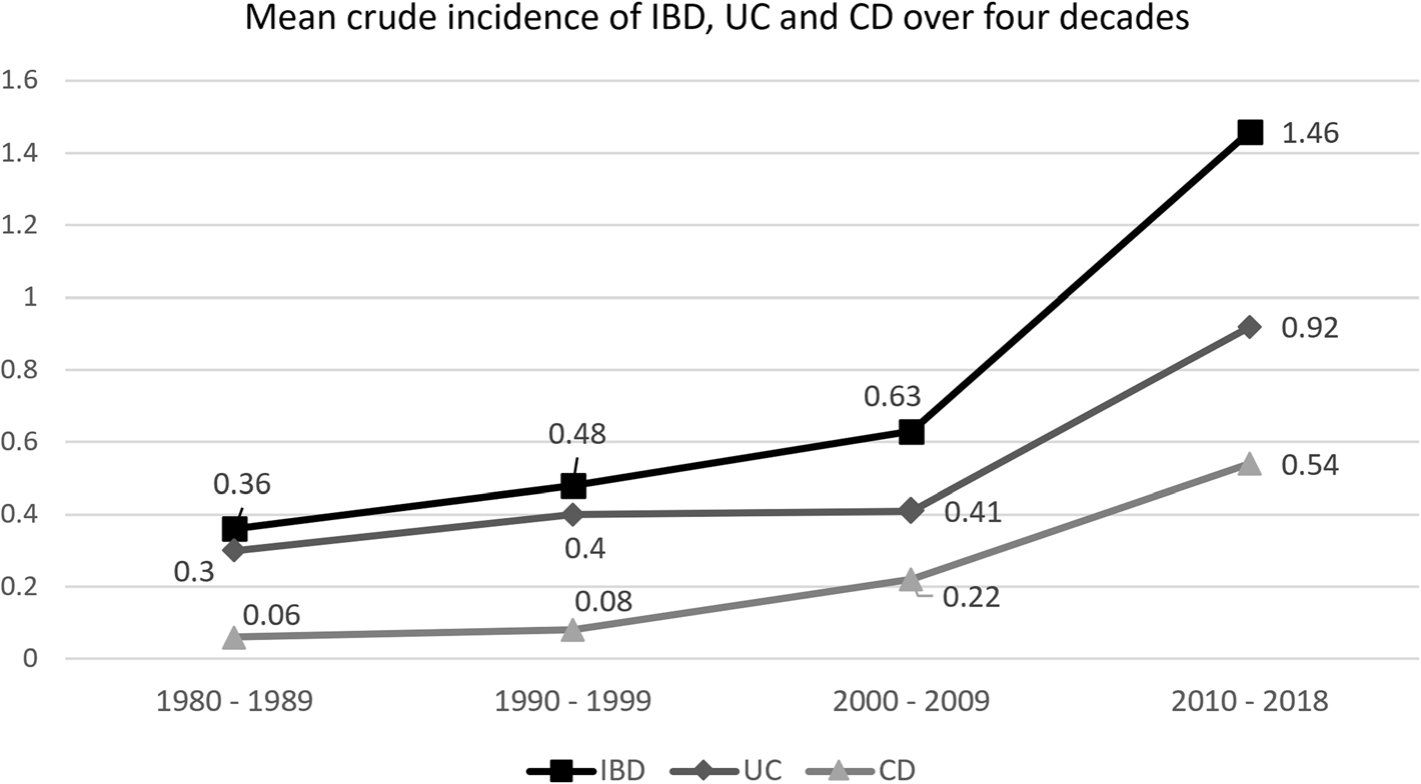
Mean crude incidence of IBD, UC and CD over four decades

A total of 281 UC and 132 CD patients (413 IBD patients) were identified. Mean crude incidence of IBD has increased steadily over the first three decades: 0.36 (1980–1989), 0.48 (1990–1999) and 0.63 per 100,000 person-years (2000–2009). In the 2010 to 2018 period, the mean crude incidence has doubled to 1.46 per 100,000 person-years. The incidence trends of UC and CD are also shown in Fig. 1. Initially, UC was much more common than CD, however, there was a significant rise in the incidence of CD from the year 2000, as depicted by reducing UC:CD ratio: 5:1 (1980–1989), 5:1 (1990–1999), 1.9:1 (2000–2009) and 1.7:1 (2010–2018) (Table 1).

**Table 1:** Mean crude incidence of IBD, UC and CD in UKMMC over the last four decades

Year	Population	IBD, N Mean crude incidence^a^	UC, N Mean crude incidence^a^	CD, N Mean crude incidence^a^	UC:CD ratio
1980	919,610	36 cases 0.36	30 cases 0.3	6 cases 0.06	5:1
1981	938,002				
1982	956,762				
1983	975,897				
1984	995,415				
1985	1,015,323				
1986	1,035,630				
1987	1,056,342				
1988	1,077,469				
1989	1,099,018				
1990	1,120,999	58 cases 0.48	48 cases 0.4	10 cases 0.08	5:1
1991	1,145,342				
1992	1,162,063				
1993	1,179,029				
1994	1,196,242				
1995	1,213,708				
1996	1,231,428				
1997	1,249,407				
1998	1,267,648				
1999	1,286,156				
2000	1,305,792	91 cases 0.63	59 cases 0.41	32 cases 0.22	1.9:1
2001	1,334,519				
2002	1,363,878				
2003	1,393,884				
2004	1,424,549				
2005	1,455,889				
2006	1,487,919				
2007	1,520,653				
2008	1,554,107				
2009	1,588,298				
2010	1,627,172	228 cases 1.46	144 cases 0.92	84 cases 0.54	1.7:1
2011	1,657,817				
2012	1,688,462				
2013	1,719,107				
2014	1,749,752				
2015	1,780,400				
2016	1,789,700				
2017	1,791,300				
2018	1,793,091				

*IBD* Inflammatory bowel disease, *UC* Ulcerative colitis, *CD* Crohn’s disease, *UKMMC* Universiti Kebangsaan Malaysia Medical Centre

^a^Per 100,000 person-years

### Prevalence of IBD

**Fig. 2:**
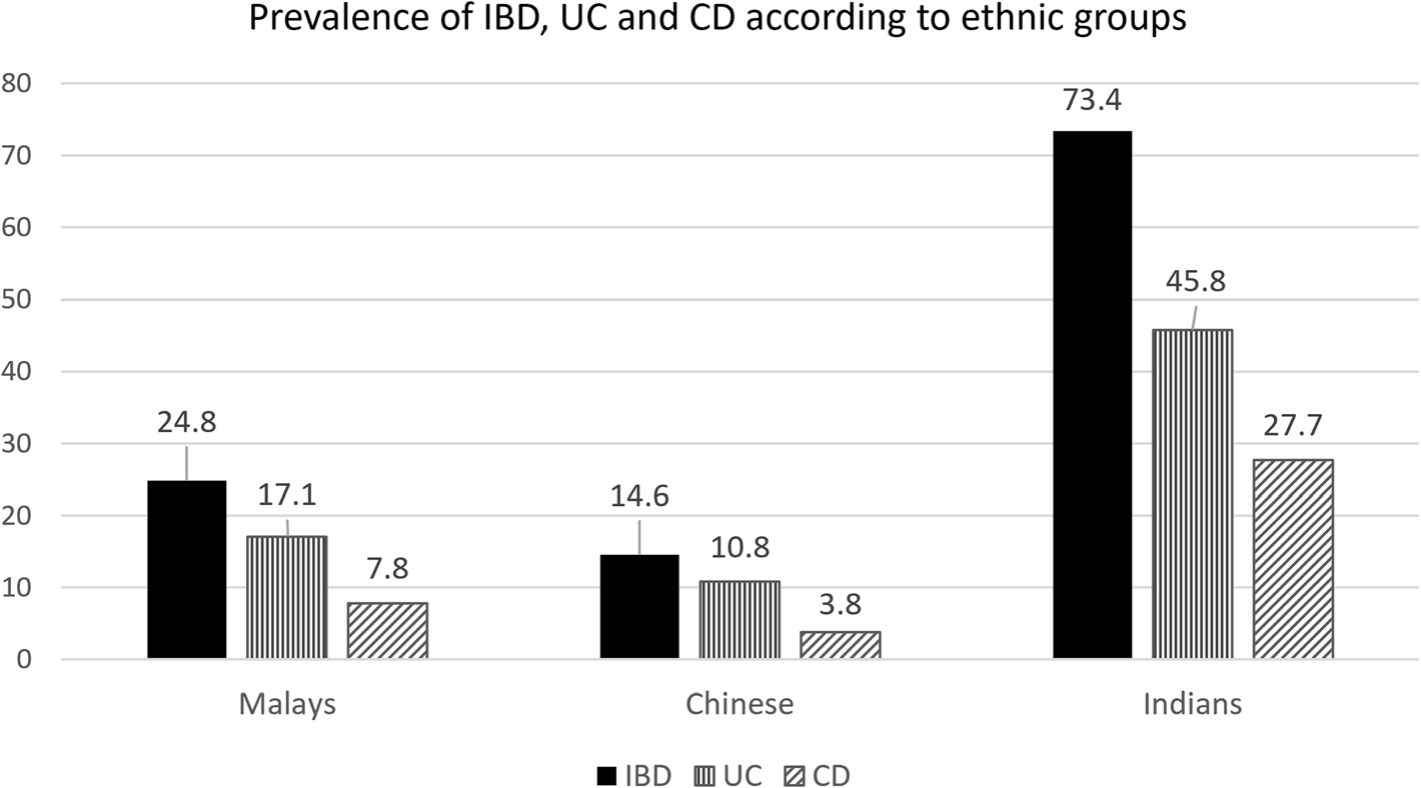
Prevalence of IBD, UC and CD according to ethnic groups

The prevalence rate of IBD, UC and CD, were 23.0, 15.67 and 7.36 per 100,000 persons respectively. When stratified according to ethnic groups, the highest prevalence of IBD was among Indians: 73.4 per 100,000 persons (UC: 45.8, CD: 27.7 per 100,000 persons), followed by Malays: 24.8 per 100,000 persons (UC: 17.1, CD: 7.8 per 100,000 persons) and Chinese: 14.6 per 100,000 persons (UC: 10.8, CD: 3.8 100,000 per persons) (Fig. 2).

### Sociodemographic characteristics of IBD patients

Of all IBD patients, 61.5% (*n* = 254) were males. UC was slightly more common in male as compared to female (male to female ratio was 1.9:1), while CD occurred equally in both (male to female ratio was 1.2:1). Most of IBD patients were non-smoker (UC: 216, 76.9%, CD: 93, 70.5%) and had tertiary education (UC: 200, 71.2%, CD: 89, 67.4%). The mean age of diagnosis was 41.2 years for UC (46.6% were middle-aged adults [36–55 years] at the time of diagnosis) and 27.4 years for CD (53.8% were young adults [19–35 years] at the time of diagnosis). More than half of the IBD patients had short duration of disease, i.e. < 5 years duration (UC: 52.6%, CD: 67.4%) (Table 2).

**Table 2:** Sociodemographic characteristics of IBD patients

Characteristics	Ulcerative Colitis *N* = 281	Crohn’s Disease *N* = 132	*P* value
Gender, *n* (%)			
Male	183 (65.1)	71 (53.8)	0.027
Female	98 (34.9)	61 (46.2)	
Ethnicity, *n* (%)			
Malay	132 (47)	60 (45.5)	0.185
Chinese	73 (26)	26 (19.7)	
Indian	76 (27)	46 (34.8)	
Smoking status, *n* (%)			
Smoker	33 (11.7)	20 (15.1)	0.375
Ex-smoker	32 (11.4)	19 (14.4)	
Non-smoker	216 (76.9)	93 (70.5)	
Education level, *n* (%)			
Primary	12 (4.3)	1 (0.8)	0.065
Secondary	69 (24.5)	42 (31.8)	
Tertiary	200 (71.2)	89 (67.4)	
Family history, *n* (%)			
IBD	2 (0.7)	5 (3.8)	0.276
CRC	3 (1.1)	2 (1.5)	
Duration of disease (Years), *n* (%)			
Short (< 5)	148 (52.6)	89 (67.4)	0.006
Long (5–10)	62 (22.1)	26 (19.7)	
Very long (> 10)	71 (25.3)	17 (12.9)	
Mean Age of Diagnosis	41.2 (+/− 16.8)	27.4 (+/− 15.9)	
Age of diagnosis, n (%)			
Adolescence (< 19)	9 (3.2)	30 (22.7)	< 0.00001
Young Adults (19–35)	88 (31.3)	71 (53.8)	
Middle –Aged Adults (36–55)	131 (46.6)	15 (11.4)	
Older Adults (> 55)	53 (18.9)	16 (12.1)	

### Disease characteristics of IBD patients

Based on Montreal’s classification, the disease extension of UC patients was as follows: 26 (9.2%) proctitis, 141 (50.2%) left sided and 114 (40.6%) extensive disease. With regards to disease location of CD patients, majority had ileo-colonic involvement (63, 47.7%), followed by colonic (38, 28.8%) and ileal involvements (30, 22.7%). Upper GI Crohn’s was rare and affecting a single patient (0.8%). Perianal Crohn’s disease occurred in 17 patients (12.9%). Observing at the behaviour of Crohn’s disease, large proportion of patients (81, 61.3%) had non-stricturing non-penetrating disease, while others developed either stricturing disease (33, 25%) or penetrating disease (10, 7.6%). Eight patients (6.1%) had concomitant stricturing and penetrating disease.

Extra intestinal manifestations of IBD were observed more in CD (71, 53.8%) as compared to UC (34, 12%), with arthropathy being the most prevalence extra intestinal manifestation in both UC:22, 64.7% and CD: 31, 43.7%. In term of disease complications, 3.2% of UC patients developed anaemia, 2.8% developed IBD-related colorectal cancer and 1.4% developed colonic dysplasia. For CD patients, 15.9% had anaemia, 32.6% complicated with fistula and 0.8% (1 patient) developed IBD-related colorectal cancer (Table 3).

**Table 3:** Disease characteristics of IBD patients

Characteristics	Ulcerative Colitis *N* = 281	Crohn’s Disease *N* = 132
Montreal’s classification		
Location, n (%)		
Proctitis (E1)	26 (9.2)	N/A
Left Sided (E2)	141 (50.2)	N/A
Extensive (E3)	114 (40.6)	N/A
Ileal (L1)	N/A	30 (22.7)
Colonic (L2)	N/A	38 (28.8)
Ileo-colonic (L3)	N/A	63 (47.7)
Upper GI (L4)	N/A	1 (0.8)
Behavior, n (%)		
Non-stricturing non-penetrating (B1)	N/A	81 (61.3)
Stricturing (B2)	N/A	33 (25)
Penetrating (B3)	N/A	10 (7.6)
Stricturing and penetrating (B2 + B3)	N/A	8 (6.1)
Perianal disease (p), *n* (%)	N/A	17 (12.9)
Extra intestinal manifestation, *n* (%)	34 (12)	71 (53.8)
Fatty liver	3	1
Primary sclerosing cholangitis	0	1
Osteoporosis	2	9
Osteopenia	5	25
Arthropathy	21	31
Juvenile ankylosing spondylitis	0	1
Erythema nodosum	1	1
Deep vein thrombosis	1	2
Uveitis	1	2
Complications, *n* (%)		
Anaemia	9 (3.2)	21 (15.9)
Fistula	N/A	43 (32.6)
Perianal fistula	N/A	26 (60)
Recto-vaginal fistula	N/A	3 (7)
Entero-cutaneous fistula	N/A	13 (30)
Ano-cutaneous fistula	N/A	1 (2.3)
Colo-cutaneous fistula	N/A	1 (2.3)
Dysplasia	4 (1.4)	0
Malignancy	8 (2.8)	1 (0.8)

### Clinical characteristics of IBD patients

Pharmacotherapy for IBD includes 5-aminosalicylic acid (5-ASA), immunomodulators and biologic therapy. Majority of UC patients remained on 5-ASA (94%) with 26% were on immunomodulators. Only 3.6% were treated with biologic therapy (nine patients received Infliximab and a single patient received Golimumab). On the other hand, 27.3% of CD patients received biologic therapy (Infliximab: 16 patients, Adalimumab: 16 patients Vedolizumab: 3 patients and Ustekinumab: 1 patient). Of all IBD patients, larger proportion of CD patients underwent IBD-related surgery as compared to UC patients (46, 34.8% vs 11, 3.9%). Approximately 43.1% of UC patients have diabetes, hypertension and dyslipidemia while lower percentage was seen in CD patients (20 out of 132 patients; 15.1%) (Table 4).

**Table 4:** Choices of treatment and comorbidity for IBD patients from 1980 until 2018

Characteristics	Ulcerative Colitis *N* = 281	Crohn’s Disease *N* = 132
Treatment, n (%)		
5-ASA	264 (94)	40 (30)
Immunomodulator	74 (26)	82 (62)
Azathioprine	73	80
Mercaptopurine (6-MP)	1	2
Steroid	7 (4.7)	8 (11.4)
Biologics	10 (3.6)	36 (27.3)
Adalimumab	0	16
Infliximab	9	16
Golimumab	1	0
Ustekinumab	0	1
Vedolizumab	0	3
Curcumin extract	0	1
No treatment	9 (3.2)	4 (3.0)
Surgery, *n* (%)	11 (3.9%)	46 (34.8)
Panproctocolectomy + ileostomy	10	N/A
Ileorectal anastomotic resection + ileostomy	1	N/A
Right hemicolectomy	N/A	10
Total colectomy	N/A	3
Right limited hemicolectomy	NA	4
Right transverse colostomy	N/A	2
Segmental colectomy	N/A	1
Small bowel resection	N/A	8
Incision and drainage for fistula	N/A	8
Seton insertion for fistula	N/A	13
Major Comorbidity, *n* (%)		
Type 2 Diabetes Mellitus	50 (17.8)	6 (4.5)
Hypertension	39 (13.9)	12 (9.1)
Dyslipidemia	32 (11.4)	2 (1.5)
Ischemic Heart Disease	8 (2.8)	1 (0.7)
Asthma	7 (2.5)	2 (1.5)
Chronic kidney disease	2 (0.7)	1 (0.7)

## Discussion

Inflammatory bowel disease is a global disease and contributes to the public health burden, although it was initially regarded as a rare disease in developing countries including Malaysia. Malaysia is a multi-racial country with three major ethnicities are Malays, Chinese and Indians, making it to be unique when dealing with the rising incidence of IBD. The incidence of IBD differs across different demographic categories, which means the clinical presentation of IBD patients is distinctive for a certain type of population. As IBD emerges in Malaysia, there are only limited number of studies that documented the trend of the IBD incidence over the last 40 years. It is important to raise awareness and better understanding in IBD for either physicians or patients resulting in new research opportunities and subsequently improved quality of life of IBD patients. Also, with this data published, there will be a reform in the IBD research which was previously less funded by the grant provider. We conducted a retrospective study aimed to reveal the incidence trends including sociodemographic and clinical characteristics of IBD in the last four decades at a tertiary referral hospital, UKMMC. Data were collected primarily from the UKMMC IBD registry. IBD registry was updated every 1 to 3 months and retained for ongoing research purposes and subsequently improved the management and care of IBD patients. The diagnostic rates of both UC and CD were indeed increasing with UC was more common than CD. However, we observed a reverse trend from the year 2000 until July 2018 with a reduction in UC to CD ratio. This depicts the emergence of CD cases in Malaysia, which resembles with the current disease pattern in certain parts of Asia including Hong Kong, Japan and Korea [14]. Environmental risk factors for example breast fed more than 12 months (aOR 0.10, 95% CI 0.04 to 0.30) and antibiotic use before the age of 15 years (aOR 0.19, 95% CI 0.07 to 0.52) were documented to be protective for the development of CD among Asians [15]. However, in this study we did not capture dietary factors and other environmental factors that may influence the incidence of CD.

Majority of UC cases were seen among male but there was no gender difference for CD. This result was dissimilar from the local data published previously by Hilmi et al., where they documented the gender difference was observed in the CD and not UC cases [11]. Previous studies postulated that the gender difference in IBD was caused by multiple factors. A study conducted among Dutch IBD patients involving 2118 CD and 1269 UC concluded that gender differences were featured based on age of disease onset, disease extent and presence of extra intestinal manifestations [16]. A meta-analysis study on the Chinese population consisted of a median number of 69 CD and 189 UC cases identified male was more predominant in both CD and UC with the median sex ratio (male to female) was 1.28 [17].

The mean age of diagnosis for UC in this study fell between 36 to 55 years with more than 40% were among middle-aged adults. While for CD, the mean age of CD fell between 19 to 35 years with more than 50% were among young adults. These observations were similar to most of the studies reported in the West and Asia countries [11, 18]. Malaysia is a multi-racial country with a population of 30 million people who practice various religions. Three major races are Malays, Indians and Chinese. Our recent data noted that IBD was predominantly noticed among Indians, followed by Malays and Chinese. A local data previously reported that IBD (both UC and CD) with limited number of patients were predominantly seen among Indians, followed by Malays and Chinese [11, 19, 20]. This finding highlighted the diagnosis of IBD which can occur among high risk groups i.e. young adults of Indian ethnicity should be made known to primary care physician so that a timely referral to the gastroenterologist can be made.

Among the recruited patients, the majority was non-smokers; which was again similar to the reported data in Malaysia [11, 19, 20]. We can’t conclude whether smoking is either a risk or protective factor among the IBD population in this region as we did not look into a non-IBD group. Based on the western population study, cigarette smoking was thought to increase the risk of CD and the opposite for UC. A recent study encompassed China and India populations as a representative for Asians failed to conclude the association between smoking and IBD [21]. Another exciting finding from this study was that most of IBD patients have tertiary education, although this was a biased population attending a tertiary hospital. The level of education attained by individuals is influenced by socioeconomic status. Based on the National Health & Morbidity survey 2015, 94% (95% CI) of Malaysian adults did not take adequate fruits and/or vegetables as recommended by the WHO [22]. The low consumption of fruits and vegetable intake may explain the higher incidence of chronic diseases including IBD in this country even among IBD patients with higher socioeconomic status [22]. In term of familial penetrance, only less than five UC patients have either family history of IBD or CRC. Similarly, less than 4% of CD patients have family history of IBD or CRC. This affirmed the lack of familial penetrance among Asians [18]. Unlike in a study investigated of more than 8000 Danish population with CD have an exponential increased risk in individuals with third-degree to first-degree relatives [23].

We used Montreal’s classification of IBD as it gives a good inter-observer agreement for the extent of disease in UC [24]. Half of the UC patients (~ 50%) was left-sided and ~ 40% has extensive disease. This finding was slightly more as compared to previous reported study which was between 37.3 to 39% [11]. Therefore, this group of patients have higher tendency to develop complications and IBD-related neoplasia in the future [25]. Almost half of the CD patients have ileo-colonic disease and three-quarters have non-stricturing, non-penetrating disease character which portrays the overall lesser aggressiveness of CD. Upper gastro-intestinal CD was reported to be rare among Asians, and our findings also echoed previous findings where only a single patient was diagnosed with isolated upper GI involvement [26]. This study also showed more than 12% of CD patients had perianal disease and about a third with fistulizing CD. After looking closely on all fistulae cases based on their ethnicities, fistulising disease was commonly seen among Indian patients.

An alarming feature of our observation in our centre was the number of UC patients who have co-morbidities associated with metabolic syndrome. The link between metabolic syndrome and IBD was described and the possible explanation was due to adipose tissue dysregulation, chronic inflammation and ineffective immune system [27]. More than three quarters of our UC-related neoplasia patients have type 2 diabetes mellitus (T2DM) which was poorly controlled at the time of neoplasia detection. Disease-linked inflammation, which is the essence that links UC, CRC and T2DM resulting in up-regulation of cytokines along with transforming growth factor beta (TGFβ), tumor necrosis factor alpha (TNFα), nuclear factor kappa-light-chain-enhancer of activated B cells (NFKB), reactive oxygen species (ROS) and other signaling molecules, consequently leading to imbalance in intestinal microbiota which contributes to the inevitable progression to neoplasia [28, 29]. Hence, understanding the consequence of T2DM which contributes to disease progression and prognosis is essential [30]. The patients should be alerted and stressed on the importance of their diabetic controls and all patients with IBD should be encouraged to screen regularly for metabolic syndrome.

Almost all of our UC patients (94%) and 30% of CD patients received 5 ASA, given its proven efficacy in IBD treatment [31]. Majority of moderate to severe disease CD patients were treated with immunomodulators as compared to the UC patients (less than 30%). Biologic agents were given to almost a third of our CD patients as this treatment was proven to be effective for the maintenance and remission of CD patients [32]. A small percentage (~ 3%) of our IBD patients did not receive any treatment for their mild disease in full remission.

Surgical treatment among IBD patients has been reduced over the years owing to early diagnosis, comprehensive guidelines, promotion of IBD medical education and a shift of care from surgeons to gastroenterologists [33]. The low surgical incidence among our UC patients can be attributed to medical therapy optimization. Almost one-third of our CD patients have underwent various forms of surgery, which was considerably low compared to the general surgical likelihood. With the emergence of anti-tumor necrosis factor agents and the usage of immunomodulators, both proven to reduce CD-related surgeries as the future of CD management is indeed evolving [33].

Long disease duration and extensive disease extent among general UC population are non-debatable risk factors for development of CRC [25]. However, it is exciting that the non-existence of family history of IBD or CRC among our 12 UC-related neoplasia patients, further affirmed that familial penetrance was lacking even among patients with the aggressive spectrum of UC in this region. It is worth investigating the possible gene dysregulation in different disease duration IBD [34].

Thus, endoscopic surveillance program for high risk IBD patients is therefore essential in IBD management. Based on European Crohn’s and colitis organization (ECCO) guidelines for UC, it is recommended that surveillance colonoscopy should be performed 8–10 years after disease onset in patients with extensive disease and 15 years in patients with left-sided [35]. Although the average duration taken for neoplasia development among our long disease duration patients was 26.91 years; early detection with a comprehensive colonoscopy surveillance program would be essential for the future of IBD-related neoplasia in this region.

Our study’s strengths include a resonably large number of sample size (*n* = 413 IBD patients), a prolonged study period (40 years) and the fact that UKMMC is a tertiary care centre for IBD in Kuala Lumpur, capital city of Malaysia. These enable us to examine the IBD incidence trends as well as to provide a more representative data on IBD patients in Malaysia. Our study limitation is mainly due to the nature of its retrospective analysis. In addition, we do not capture any data on dietary factors that might be relevant as a risk or protective factor for IBD. This could open up more opportunities for future research in investigating possible environmental risk factors such as dietary intake and life style especially when there is lack of genetic susceptibility among IBD patients in this Asia region.

## Conclusion

This four-decade study concludes that there is emerging trend of IBD in Kuala Lumpur and prevailed mostly among Indians followed Malays and Chinese. The clinical characteristics among these patients were males, non-smokers, highly educated, diagnosed at young age and negative family history of IBD.

## Abbreviations

5-ASA: 5-aminosalicylic acid
CD: Crohn’s disease
GI: Gastrointestinal
IBD: Inflammatory bowel disease
MRI: Magnetic resonance imaging
NFKB: Nuclear factor kappa-light-chain-enhancer of activated B cells
ROS: Reactive oxygen species
RR: Risk ratio
SPSS: Statistical Package for Social Sciences
T2DM: Type 2 diabetes mellitus
TGFβ: Transforming growth factor beta
TNFα: Tumor necrosis factor alpha
UC: Ulcerative colitis
UKMMC: Universiti Kebangsaan Malaysia Medical Centre
